# Clinical Impact of Primary Tumor Location in Metastatic Colorectal Cancer Patients Under Later-Line Regorafenib or Trifluridine/Tipiracil Treatment

**DOI:** 10.3389/fonc.2021.688709

**Published:** 2021-06-15

**Authors:** Hiromichi Nakajima, Shota Fukuoka, Toshiki Masuishi, Atsuo Takashima, Yosuke Kumekawa, Takeshi Kajiwara, Kentaro Yamazaki, Yuji Negoro, Masato Komoda, Akitaka Makiyama, Tadamichi Denda, Yukimasa Hatachi, Takeshi Suto, Naotoshi Sugimoto, Masanobu Enomoto, Toshiaki Ishikawa, Tomomi Kashiwada, Koji Ando, Satoshi Yuki, Hiroyuki Okuyama, Hitoshi Kusaba, Daisuke Sakai, Koichi Okamoto, Takao Tamura, Kimihiro Yamashita, Masahiko Gosho, Toshikazu Moriwaki

**Affiliations:** ^1^ Department of Gastrointestinal Oncology, National Cancer Center Hospital East, Chiba, Japan; ^2^ Division of Cancer Immunology, Exploratory Oncology Research and Clinical Trial Center, National Cancer Center, Chiba, Japan; ^3^ Department of Clinical Oncology, Aichi Cancer Center Hospital, Aichi, Japan; ^4^ Gastrointestinal Medical Oncology Division, National Cancer Center Hospital, Tokyo, Japan; ^5^ Department of Gastroenterology, Saitama Cancer Center, Saitama, Japan; ^6^ Department of Gastrointestinal Medical Oncology, National Hospital Organization Shikoku Cancer Center, Ehime, Japan; ^7^ Division of Gastrointestinal Oncology, Shizuoka Cancer Center, Shizuoka, Japan; ^8^ Clinical Oncology Division, Kochi Health Sciences Center, Kochi, Japan; ^9^ Department of Gastrointestinal and Medical Oncology, National Hospital Organization Kyushu Cancer Center, Fukuoka, Japan; ^10^ Department of Hematology/Oncology, Japan Community Healthcare Organization Kyushu Hospital, Fukuoka, Japan; ^11^ Cancer Center, Gifu University Hospital, Gifu, Japan; ^12^ Division of Gastroenterology, Chiba Cancer Center, Chiba, Japan; ^13^ Department of Clinical Oncology, Kansai Rosai Hospital, Hyogo, Japan; ^14^ Department of Gastroenterological Surgery, Yamagata Prefectural Central Hospital, Yamagata, Japan; ^15^ Department of Medical Oncology, Osaka International Cancer Institute, Osaka, Japan; ^16^ Department of Gastrointestinal and Pediatric Surgery, Tokyo Medical University, Tokyo, Japan; ^17^ Department of Specialized Surgeries, Graduate School of Medicine and Dentistry, Tokyo Medical and Dental University, Tokyo, Japan; ^18^ Division of Hematology, Respiratory Medicine and Oncology, Department of Internal Medicine, Faculty of Medicine, Saga University, Saga, Japan; ^19^ Department of Surgery and Science, Graduate School of Medical Sciences, Kyushu University, Fukuoka, Japan; ^20^ Department of Gastroenterology and Hepatology, Hokkaido University Hospital, Hokkaido, Japan; ^21^ Department of Clinical Oncology, Faculty of Medicine, Kagawa University, Kagawa, Japan; ^22^ Department of Medicine and Biosystemic Science, Kyushu University Graduate School of Medical Sciences, Kyushu University, Fukuoka, Japan; ^23^ Department of Frontier Science for Cancer and Chemotherapy, Osaka University Graduate School of Medicine, Osaka, Japan; ^24^ Department of Surgery, National Defense Medical College, Saitama, Japan; ^25^ Department of Medical Oncology, Faculty of Medicine, Kindai University, Osaka, Japan; ^26^ Division of Gastrointestinal Surgery, Department of Surgery, Graduate School of Medicine, Kobe University, Hyogo, Japan; ^27^ Department of Biostatistics, Faculty of Medicine, University of Tsukuba, Ibaraki, Japan; ^28^ Department of Gastroenterology, Faculty of Medicine, University of Tsukuba, Ibaraki, Japan

**Keywords:** regorafenib, trifluridine/tipiracil, colorectal cancer, primary tumor location, biomarker

## Abstract

**Background:**

Primary tumor location (PTL) is an important prognostic and predictive factor in the first-line treatment of metastatic colorectal cancer (mCRC). Although regorafenib (REG) and trifluridine/tipiracil (FTD/TPI) have been introduced recently, the clinical impact of PTL in these treatments is not well understood.

**Materials and Methods:**

We retrospectively evaluated patients with mCRC who were registered in a multicenter observational study (the REGOTAS study). The main inclusion criteria were Eastern Cooperative Oncology Group performance status (ECOG PS) of 0–2, refractory or intolerant to fluoropyrimidines, oxaliplatin, irinotecan, angiogenesis inhibitors, anti-epidermal growth factor receptor therapy (if RAS wild-type), and no prior use of REG and FTD/TPI. The impact of PTL on overall survival (OS) was evaluated using Cox proportional hazard models based on baseline characteristics.

**Results:**

A total of 550 patients (223 patients in the REG group and 327 patients in the FTD/TPI group) were included in this study, with 122 patients with right-sided tumors and 428 patients with left-sided tumors. Although the right-sided patients had significantly shorter OS compared with the left-sided patients by univariate analysis (*p* = 0.041), a multivariate analysis revealed that PTL was not an independent prognostic factor (hazard ratio, 0.95; *p* = 0.64). In a subgroup analysis, the OS was comparable between the REG and FTD/TPI groups regardless of PTL (*p* for interactions = 0.60).

**Conclusions:**

In the present study, PTL is not a prognostic and predictive factor in patients with mCRC under later-line REG or FTD/TPI therapy.

## Introduction

The standard of care for patients with metastatic colorectal cancer (mCRC) has evolved with combination chemotherapy regimens, including cytotoxic agents (e.g., fluoropyrimidine [FU], oxaliplatin [OX], and irinotecan [IRI]), angiogenesis inhibitors (e.g., bevacizumab, aflibercept, and ramucirumab), and anti-epidermal growth factor receptor (EGFR) antibodies (e.g., cetuximab, and panitumumab) for patients with RAS wild-type tumors. (1–8) In recent years, regorafenib (REG) and trifluridine/tipiracil (FTD/TPI) significantly improved the overall survival (OS) in patients with chemorefractory mCRC compared with placebo (9–12) and have been available in clinical practice.

Accumulating evidence indicates that primary tumor location (PTL) is an important prognostic factor in mCRC, as right-sided tumors are associated with poorer outcomes than left-sided tumors, especially after first-line treatments (13–17). Retrospective analyses of randomized trials in first-line settings indicate that right-sided primary tumors were negative predictive markers for the efficacy of anti-EGFR therapy. (13, 14) Therefore, anti-EGFR-based first-line treatment was only recommended for patients with left-sided primary tumors in several international guidelines. (15–17) Thus, treatment stratification based on PTL is one of the critical aspects of standard care for mCRC.

However, the clinical impact of PTL in patients with mCRC under later-line REG or FTD/TPI treatment is not well understood. Although a subgroup analysis of these pivotal trials showed a survival benefit of REG and FTD/TPI regardless of PTL, (9, 11, 12) no randomized study has compared REG and FTD/TPI directly. Thus, the optimal treatment sequence of REG and FTD/TPI according to PTL remains unclear.

We previously reported that the multicenter, large cohort, and observational REGOTAS study showed no significant difference in OS between REG and FTD/TPI treatments in patients with mCRC. (18) The present study investigated the prognostic and predictive values of PTL in mCRC patients under later-line REG and FTD/TPI treatment in the REGOTAS study.

## Materials and Methods

### Patients

The present study retrospectively examined the clinical records of patients with mCRC treated with later-line REG or FTD/TPI chemotherapy during the period from June 1, 2014, to November 30, 2015. All the patients were registered in the REGOTAS study, which is described in detail elsewhere. (18) The main eligibility criteria were as follows: (1) histologically confirmed colorectal adenocarcinoma; (2) no prior treatment using REG and FTD/TPI; (3) previous treatment with FU, OX, IRI, bevacizumab, and anti-EGFR antibody (in patients with RAS wild-type tumor); (4) Eastern Cooperative Oncology Group performance status (ECOG PS) of 0–2; and (5) adequate organ function. Patients who could receive only a specific drug treatment, either REG or FTD/TPI, due to comorbidity and/or medical history. The primary tumors were classified as right-sided tumors if located between the cecum and the splenic flexure of the transverse colon. Others, from the descending colon to the rectum, were defined as left-sided tumors.

The present study was approved by the ethics committees at each institution and was in accordance with the guidelines for biomedical research specified in the Declaration of Helsinki. The REGOTAS study was registered with the University Medical Information Network (number UMIN000020416). The requirement for informed consent was waived due to the retrospective nature of this study.

### Statistical Analysis

The exploratory primary endpoint was OS, defined as the time from the start of REG or FTD/TPI treatment to death or last follow-up. The following pretreatment clinical data and baseline laboratory values were used in the analysis as covariates: age, sex, body mass index, ECOG PS, surgery on primary tumor, histological grade, RAS status, metastatic tumor site (liver metastasis, lung metastasis, lymph node metastasis, and peritoneal dissemination), number of metastatic organ sites, and treatment duration from initiation of first-line chemotherapy.

The Mann-Whitney U test was used to compare the continuous variables, and Fisher’s exact test to compare the categorical variables. Survival curves were estimated using the Kaplan-Meier method, and differences between the groups were analyzed with the log-rank test. Hazard ratios (HRs) were estimated using the Cox proportional hazard model. OS was analyzed using univariate and multivariate Cox regression analyses. The backward selection method was performed to select covariates retained (*p* < 0.1) in the multivariate analysis.

Primary analysis was conducted using all patients with sufficient information. A 1:1 matching using the propensity score (the propensity-score-matched cohort) was performed as a sensitivity analysis. The details of the propensity-score-matched cohort were described elsewhere. (18) All *p* values < 0.05 were considered statistically significant. All statistical analyses were performed using EZR (Saitama Medical Center, Jichi Medical University, Saitama, Japan), which is a graphical user interface for R (The R Foundation for Statistical Computing).

## Results

### Patients

**Figure 1:**
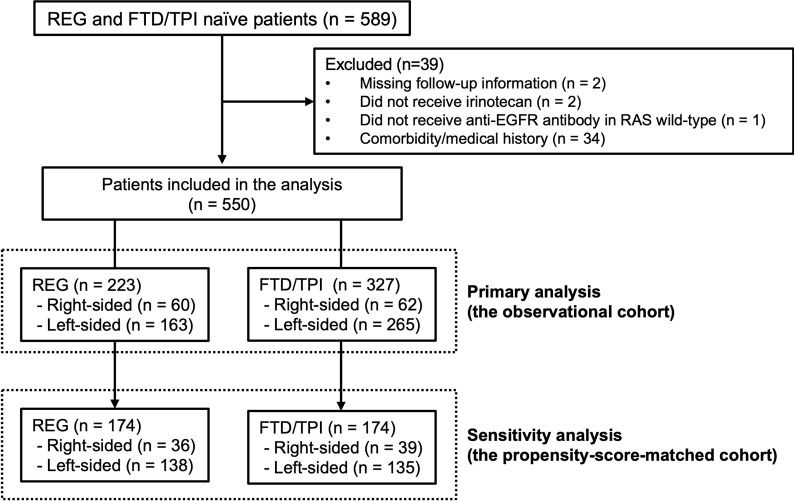
Patient selection flow diagram.

Among 589 mCRC patients, 550 met the inclusion criteria (the observational cohort), including 223 patients in the REG group and 327 patients in the FTD/TPI group (**Figure 1**). Sixty patients (27%) in the REG group and 62 patients (19%) in the FTD/TPI group had right-sided tumors (*p* = 0.029). Patient characteristics are summarized in **Table 1**. More patients with right-sided tumors had lower BMI, RAS mutations, lung metastases, and less than three prior lines of chemotherapy than those with left-sided tumors in both the REG and FTD/TPI groups. The patients’ follow-up was until September 2016. The median follow-up at the time of analysis was 17.2 months, and 418 (76%) patients had died at the time of analysis.

**Table 1:** Patient characteristics.

	REG group		*p* value*	FTD/TPI group		*p* value*
	Right (n = 60)	Left (n = 163)		Right (n = 62)	Left (n = 265)	
**Age, years**						
Median (IQR)	65 [58–71]	64 [55–71]	0.30	65 [59–72]	64 [55–70]	0.17
≥ 65, n (%)	31 (51.7)	76 (46.6)	0.55	33 (53.2)	123 (46.4)	0.40
**Sex, n (%)**			0.17			0.89
Male	29 (48.3)	97 (59.5)		38 (61.3)	159 (60.0)	
Female	31 (51.7)	66 (40.5)		24 (38.7)	106 (40.0)	
**BMI, n (%)**			0.025			0.046
≥ 18.5	47 (78.3)	147 (90.2)		45 (72.6)	222 (83.8)	
**ECOG PS, n (%)**			0.086			0.41
PS0 or 1	56 (93.3)	160 (98.2)		56 (90.3)	248 (93.6)	
PS2	4 (6.7)	3 (1.8)		6 (9.7)	17 (6.4)	
**Surgery on primary tumor, n (%)**			0.20			0.31
Yes	51 (85.0)	125 (76.7)		45 (72.6)	210 (79.2)	
**Histological grade, n (%)**			0.01			0.35
Well/mod	48 (80.0)	149 (91.4)		55 (88.7)	242 (91.3)	
Others	10 (16.7)	7 (4.3)		3 (4.8)	16 (6.0)	
Missing	2 (3.3)	7 (4.3)		4 (6.5)	7 (2.6)	
**RAS status, n (%)**			< 0.001			0.013
Mutant	42 (70.0)	67 (41.1)		37 (59.7)	124 (46.8)	
Missing	0 (0.0)	6 (3.7)		3 (4.8)	3 (1.1)	
**Metastasis, n (%)**						
Liver	40 (66.7)	101 (62.0)	0.54	40 (64.5)	161 (60.8)	0.66
Lung	29 (48.3)	51 (31.3)	0.027	28 (45.2)	79 (29.8)	0.024
Lymph node	26 (43.3)	68 (41.7)	0.88	21 (33.9)	122 (46.0)	0.089
Peritoneum	15 (25.0)	20 (12.3)	0.036	26 (41.9)	41 (15.5)	< 0.001
**Number of metastatic organ site(s), n (%)**			0.17			
≥ 3	11 (18.3)	46 (28.2)		25 (40.3)	103 (38.9)	0.89
**Duration from initiation of 1st line chemotherapy, n (%)**			0.13			0.078
≥ 18 months	39 (65.0)	124 (76.1)		40 (64.5)	201 (75.8)	
**Prior regimens, n (%)**			0.024			< 0.001
≥ 3	21 (35.0)	85 (52.1)		17 (27.4)	147 (55.5)	

*The p values were calculated using the Mann-Whitney U test for continuous variable and Fisher’s exact probability test for categorical variables.

BMI, body mass index; ECOG PS, Eastern Cooperative Oncology Group performance status; IQR, interquartile range; RAS, rat sarcoma; REG, regorafenib; FTD/TPI, trifluridine/tipiracil.

### Efficacy

#### Prognostic Value of PTL

In the observational cohort (the REG and FTD/TPI groups), the median OS was 5.9 months (95% CI 5.3–7.1) in the right-sided tumors and 8.0 months (7.3–9.1) in the left-sided tumors (unadjusted HR 0.79 [95% CI 0.63–0.99], log-rank *p* = 0.041; **Supplemental Figure 1A**). The subgroup analysis of each treatment group also demonstrated that the OS was shorter in right-sided tumors (**Supplemental Figures 1B, C**). **Table 2** showed the results of univariate and multivariate analyses of OS. Multivariate analysis revealed that PTL was not significantly associated with OS (adjusted HR 0.95, [95% CI 0.75–1.20], *p* = 0.64).

**Table 2:** Univariate and multivariate analyses of overall survival (OS) in the observational cohort.

Variable	Category	Univariate	*p* value*	Multivariate	*p* value*
		HR (95% CI)		HR (95% CI)	
**PTL**	Left *vs.* Right	0.79 (0.63–0.99)	0.042	0.95 (0.75–1.20)	0.64
**Treatment group**	FTD/TPI *vs.* REG	1.02 (0.84–1.25)	0.80		
**Age**	≥ 65 *vs.* < 65	1.22 (1–1.48)	0.044	1.32 (1.08–1.61)	< 0.001
**Sex**	Female *vs.* Male	1.05 (0.86–1.27)	0.63		
**BMI**	≥ 18.5 *vs.* 18.5	0.94 (0.72–1.22)	0.62		
**ECOG PS**	PS2 v*s.* PS1 or 2	1.48 (0.99–2.21)	0.059	1.57 (1.03–2.39)	0.036
**Surgery on primary resection**	Yes *vs.* No	0.60 (0.48–0.76)	< 0.001	0.74 (0.58–0.94)	0.014
**Histology**	Others *vs.* well/mod	1.03 (0.68–1.56)	0.87		
**RAS status**	Mutant *vs.* Wild	1.18 (0.99–1.41)	0.067	1.10 (0.91–1.33)	0.33
**Liver metastasis**	Yes *vs.* No	1.65 (1.35–2.03)	< 0.001	1.59 (1.252.01)	< 0.001
**Lymph node metastasis**	Yes *vs.* No	1.40 (1.15–1.7)	< 0.001	1.34 (1.03–1.73)	0.026
**Lung metastasis**	Yes *vs.* No	0.84 (0.69–1.03)	0.089	0.92 (0.72–1.18)	0.52
**Peritoneal metastasis**	Yes *vs.* No	1.52 (1.2–1.93)	< 0.001	1.52 (1.13–2.04)	0.0051
**Number of metastatic organ site(s)**	≥ 3 *vs.* < 3	1.57 (1.28–1.92)	< 0.001	1.10 (0.80–1.52)	0.55
**Duration from initiation of 1st line chemotherapy**	≥ 18 months *vs.* < 18 months	0.63 (0.51–0.78)	< 0.001	0.65 (0.52–0.81)	< 0.001
**Prior regimens**	≥ 3 *vs.* < 3	0.85 (0.7–1.03)	0.11		

^＊^p values were calculated using the Cox proportional-hazards model.

BMI, body mass index; ECOG PS, Eastern Cooperative Oncology Group performance status; RAS; rat sarcoma; PTL, primary tumor location; REG, regorafenib; FTD/TPI, trifluridine/tipiracil.

#### Predictive Value of PTL

**Figure 2:**
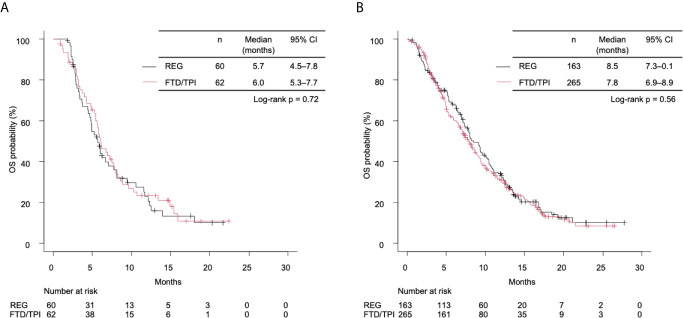
**(A)** Kaplan-Meier curves of overall survival (OS) stratified by treatment group in right-sided tumors. The median OS times of the REG and FTD/TPI groups were 5.7 months (95% CI 4.5–7.8) and 6.0 months (5.3–7.7), respectively (log-rank *p* = 0.72). **(B)** Kaplan-Meier curves of OS stratified by treatment for left-sided tumors. The median OS times of the REG and the FTD/TPI groups were 8.5 months (95% CI 7.3–10.1) and 7.8 months (6.9–8.9), respectively (log-rank *p* = 0.56). REG, regorafenib; FTD/TPI, trifluridine/tipiracil.

In the right-sided tumors, the median OS was 5.7 months (4.5–7.8) in the REG group and 6.0 months (5.3–7.7) in the FTD/TPI group (unadjusted HR 0.93 [95% CI 0.62–1.39], log-rank *p* = 0.71; **Figure 2A**). In the left-sided tumors, the median OS was 8.5 months (7.3–10.2) in the REG group and 7.8 months (6.9–8.9) in the FTD/TPI group (unadjusted HR 1.07 [95% CI 0.85–1.34], log-rank *p* = 0.56; **Figure 2B**). Interactions between treatment groups and PTL were not significant (*p* for interactions = 0.60). In the right-sided tumors, the progression-free survival (PFS) tended to be longer in the FTD/TPI group (unadjusted HR 0.71 [95% CI 0.48–1.05], log-rank *p* = 0.086; **Supplemental Figure 2A**), while in the left-sided tumors, the result was comparable between the treatment groups (unadjusted HR 1.05 [95% CI 0.85–1.29], log-rank *p* = 0.64; **Supplemental Figure 2B**). The interactions between the treatment groups and PTL were not significant (*p* for interactions = 0.072). Among patients with target lesions (112 patients in the right-sided tumors and 407 patients in the left-sided tumor), no complete responses were observed, and partial response was found in 3 patients who received FTD/TPI in the left-sided tumors. The disease control rate was comparable between the treatment groups in each PTL (**Supplemental Table 1**).

#### Sensitivity Analysis

A total of 174 patients per treatment group were matched by propensity score. The details of this cohort were described in the previous report. (18) Multivariate analysis revealed that PTL was not an independent prognostic factor (adjusted HR 0.97, [95% CI 0.72–1.33], *p* = 0.87; **Supplemental Table 2**). In the subgroup analysis, the OS and PFS were similar between the treatment groups regardless of PTL (**Supplemental Figures 3A, B**). Moreover, there were no significant interactions between the treatment groups and PTL in OS and PFS (*p* for interactions = 0.82 and 0.37, respectively).

## Discussion

To the best of our knowledge, this is the first study to assess PTL as a prognostic or predictive factor during later-line REG and FTD/TPI treatments in patients with chemorefractory mCRC. As described above, there were several differences in patient characteristics according to PTL, such as RAS status and lung metastasis incidence. Nevertheless, PTL was not an independent prognostic factor in the multivariate analysis in the cohort treated with REG or FTD/TPI. Moreover, no interactions were observed between the treatment groups and PTL in terms of OS and PFS, which suggests that the efficacy of REG and FTD/TPI is not influenced by PTL.

Recent investigations revealed differences in epidemiological, clinical, and molecular-pathological profiles between the right-sided (between the cecum and transverse colon) and left-sided tumors (between the descending colon and rectum), (19–21) and patients with right-sided tumors had poorer survival than patients with left-sided tumors. (13, 14, 22, 23) However, most of the evidence on the prognostic value of PTL was based on first-line data, and few later-line data are available. In *post hoc* analyses of data from phase III studies evaluating the efficacy of later-line panitumumab, RAS wild-type patients with right-sided tumors had significantly shorter OS and PFS than those with left-sided tumors, while no clear prognostic impact of PTL was found in RAS mutant patients. (24) By contrast, in the large-scale, prospective, observational study (CORRELATE), the REG treatment outcome was comparable across the different PTLs, similar to our results. (25) Although the reasons for the different outcomes according to PTL remain unclear, different molecular profiles related to sensitivity or resistance to anti-EGFR antibodies could be responsible. (26, 27) In the CORRELATE and our study, most patients with RAS wild-type had already been treated with anti-EGFR therapy. A possible explanation for the difference in the prognostic value of PTL among studies is whether the anti-EGFR therapy-naive and RAS wild-type/left-sided patients, who would benefit more from anti-EGFR therapy, were included or not.

In the pivotal trials of REG and FTD/TPI, subgroup analyses of PTL have been reported only according to the classification of the colon and rectum. In the CORRECT trial, which compared REG with placebo, the HR for OS was 0.70 in the colon group and 0.95 in the rectum group. (9) By contrast, in the RECOURSE trial, which compared FTD/TPI with placebo, the HR for OS was 0.68 in the colon group and 0.64 in the rectum group. (11) Although these results were seemingly considered less survival benefits of REG in patients with rectal cancers, the HRs for PFS were similar between the colon and rectum groups (0.55 *vs* 0.45); therefore, we speculated that the clinical benefits of REG and FTD/TPI are similar regardless of the colon or rectum. In fact, our results support the hypothesis that the classification of the PTL had no predictive value in later-line treatment with REG or FTD/TPI.

To date, novel molecular biomarkers that predict the effectiveness of REG and FTD/TPI have been investigated. Small studies suggest that APC mutations or FGFR1 amplification in tumor tissue were more enriched in REG patients with a clinical benefit than those without, (28) and plasma VCAM-1 was potentially predictive of OS benefit in REG treatment. (29) A survival benefit of FTD/TPI was observed regardless of KRAS status. (11, 12) High thymidine kinase 1 (TK1) expression level correlated with a larger survival benefit in FTD/TPI treatment, (30) although no significant difference in TKI expression according to PTL was reported. Moreover, evolving technologies of liquid biopsies using circulating tumor DNA (ctDNA) and circulating tumor cells (CTCs) analysis have accelerated research on the dynamism of clonal evolution, enabling us to reveal molecular profiling, monitor clonal dynamics, and identify resistance mechanism by longitudinal biopsies (31–33). The subgroup and exploratory biomarker analyses in the CORRECT trial suggested that a survival benefit was observed regardless of RAS or PIK3CA mutational status in ctDNA. (34) Amatu et al. reported baseline and dynamic circulating methylated DNA as prognostic and predictive in patients treated with REG (35). The TACT-D trial (NCT03844620) is currently conducting to validate changes in ctDNA to predict resistance early and limit toxicities in mCRC patients who receive REG or FTD/TPI. More comprehensive molecular analyses in a larger cohort of patients treated with REG or FTD/TPI may be needed to clarify the exact biomarkers to predict outcomes.

It is essential to describe the limitations of this observational study. First, this is not a randomized study to directly compare REG and FTD/TPI. Treatment selection was mainly based on the patient’s request or investigator’s decision as previously described (18), which led to an inherent bias. The proportion of patients with right-sided tumors was higher in the REG than in the FTD/TPI group. The exact reasons for treatment selection were not collected in the study, but FTD/TPI may be more favored in patients with skin toxicity due to previous anti-EGFR therapies, which are used for longer in patients with left-sided tumors in early treatment settings. Second, all patients enrolled in this study were Japanese. However, the absence of ethnic differences in the analysis of the efficacy of REG and FTD/TPI in phase III trials could enable the results to be applied to all patients regardless of ethnicity. (9–12) Third, death events were observed in 76% of patients, but the follow-up period might have been relatively short. Finally, biomarkers other than RAS status (e.g., BRAF and microsatellite instability) and detailed clinical outcomes of previous treatments were not collected in this study. These limitations encourage us to conduct a prospective study with sufficient statistical power to confirm the findings of this study.

## Conclusions

Our multicenter retrospective study revealed that PTL is not a prognostic factor in patients with mCRC under later-line REG or FTD/TPI treatment. No significant difference in OS was observed between the REG and FTD/TPI groups, irrespective of PTL. Our findings highlight the importance of selecting later-line treatments regardless of PTL for patients with mCRC.

## Data Availability Statement

The raw data supporting the conclusions of this article will be made available by the authors, without undue reservation.

## Ethics Statement

The studies involving human participants were reviewed and approved by the ethics committees at each institution. Written informed consent for participation was not required for this study in accordance with the national legislation and the institutional requirements.

## Author Contributions

Concept/design: HN, SF, and TMo. Provision of study materials or patients: SF, TMa, AT, YK, TaK, KeY, YN, MK, AM, TD, YH, TS, NS, ME, TI, ToK, KA, SY, HO, HK, DS, KO, TT, KiY, and TMo. Analysis and interpretation of data: HN, SF, TMo, and MG. Manuscript writing: HN, SF, and TMo. All authors contributed to the article and approved the submitted version.

## Conflict of Interest

TM has received research funding from MSD, Daiichi-Sankyo, and has received honoraria from Ono, Takeda, Chugai, Merck Biopharma, Taiho, Bayer Yakuhin, Lilly Japan, Yakult Honsha, Ono Pharmaceutical, Bristol-Myers Squibb, and Sanofi. AT has received research funding from Ono Pharmaceutical, Takeda, MSD, Eisai, Bayer Yakuhin, Bristol-Myers Squibb, and has received honoraria from Ono Pharmaceutical, Takeda, Lilly Japan, Taiho Pharmaceutical, Chuga, Merck Biopharma. TK has received honoraria from Taiho Pharmaceutical, Chugai, Bristol-Myers Squibb, and Merck Biopharma. KY has received research funding from Taiho Pharmaceutical, and has received honoraria from Taiho Pharmaceutical, Daiichi-Sankyo, Lily Japan, Yakult Honsha, Merck Serono, Bristol-Myers Squibb, Ono Pharmaceutical, MSD, Sanofi, Chugai, Takeda, and Bayer Yakuhin. MK has received honoraria from Lily Japan. AM has received honoraria from Lilly Japan, Taiho Pharmaceutical, Ono Pharmaceutical, Bristol-Myers Squibb, and Daiichi-Sankyo. TD has received research funding from Ono Pharmaceutical, MSD, and has received honoraria from SAWA Pharmaceutical, and Sysmex. NS has research funding from MSD, Ono Pharmaceutical, Taiho, Daiichi-Sankyo, Dainippon-Sumitomo, Chugai, Beigene, and Solasia. TI has received honoraria from Taiho Pharmaceutical, Chugai, Merck Biopharma, Daiichi-Sankyo, Sanofi, Bayer Yakuhin, and Lilly Japan. SY has received honoraria from Chugai, Lilly Japan, Takeda, Bayer Yakuhin, Bristol-Myers Squibb, Taiho Pharmaceutical, MSD, Ono Pharmaceutical, Medical & Biological Laboratories, Yakult Honsha, Merck Biopharma, and Sanofi. DS has received research funding from Chugai, Daiichi-Sankyo, Lilly Japan, Yakult Honsha, Ono Pharmaceutical, Astellas Pharma, Incyte, Taiho Pharmaceutical, and Eisai, and has received honoraria from Chugai. TT has received research funding from Takeda, Chugai, Taiho Pharmaceutical, Beigene, Ono Pharmaceutical, and has received honoraria from Daiichi-Sankyo. TM has research funding from Taiho Pharmaceutical, Yakult Honsha, and has received honoraria from Taiho Pharmaceutical, Yakult Honsha, MSD, Eisai, and has received honoraria from Takeda, Chugai, Sanofi, Ono, Bristol, and Bayer Yakuhin.

The remaining authors declare that the research was conducted in the absence of any commercial or financial relationships that could be construed as a potential conflict of interest.
