# Supplementary material for: Clinical Impact of Primary Tumor Location in Metastatic Colorectal Cancer Patients Under Later-Line Regorafenib or Trifluridine/Tipiracil Treatment
Source: Front Oncol. 2021 Jun 15;11:688709. doi: 10.3389/fonc.2021.688709 (PMC8239287; doi:10.3389/fonc.2021.688709)
Supplement: Supplementary file 1 [file Image_1.pdf]

Supplemental Figure 1A

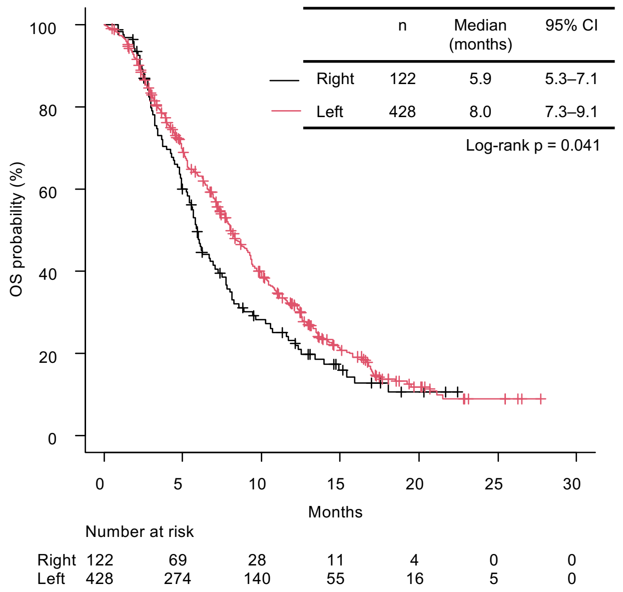

Supplemental Figure 1B

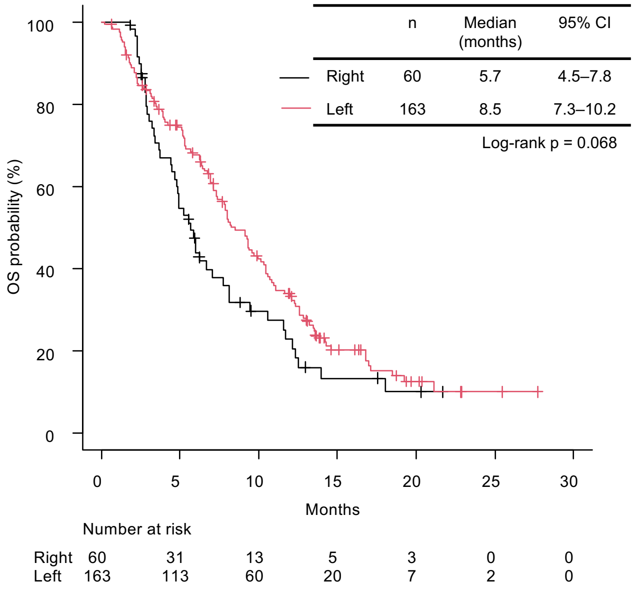

Supplemental Figure 1C

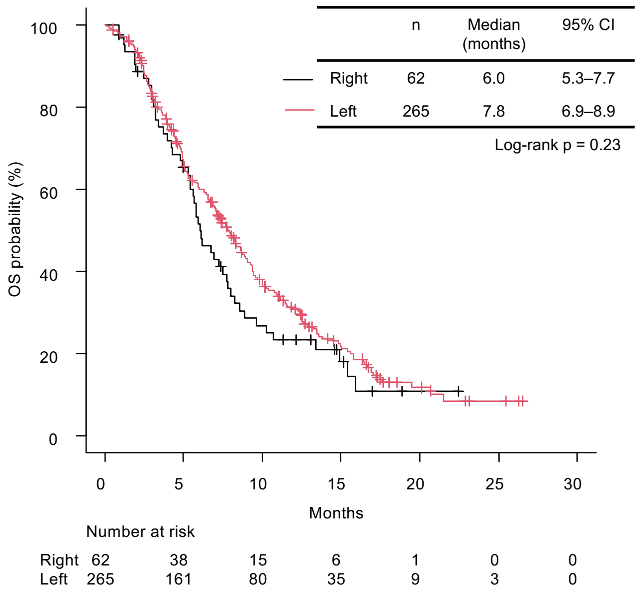

Supplemental online Figure 1. (A) Kaplan-Meier curves of overall survival (OS) stratified by primary tumor location (PTL) in the observational cohort. (B) Kaplan-Meier curves of OS stratified by PTL in the REG group. (C) Kaplan-Meier curves of OS stratified by PTL in the FTD/TPI group. REG, regorafenib; FTD/TPI, trifluridine/tipiracil

Supplemental Figure 2A

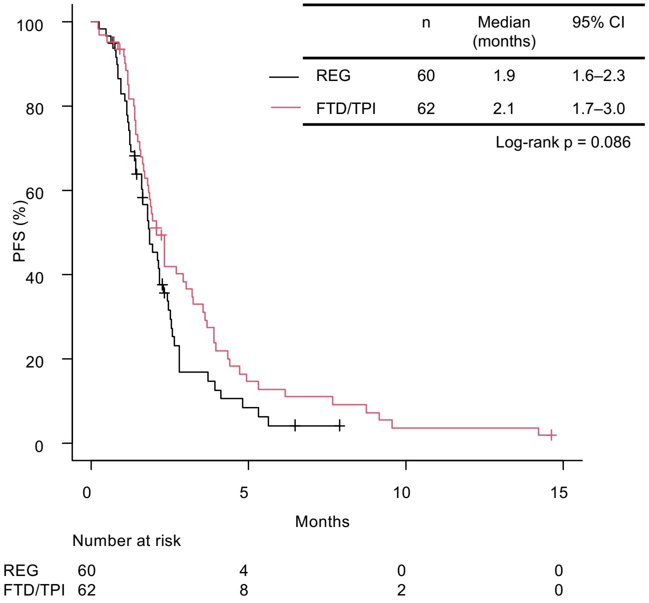

Supplemental Figure 2B

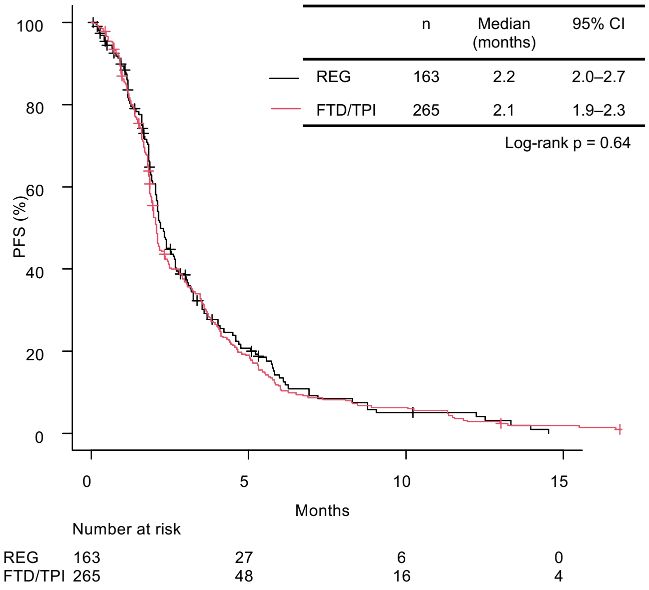

Supplemental Figure 2. (A) Kaplan-Meier curves of progression-free survival (PFS) stratified by treatment group in the right-sided tumors. (B) Kaplan-Meier curves of PFS stratified by treatment for left-sided tumors. REG, regorafenib; FTD/TPI, trifluridine/tipiracil

Supplemental Figure 3A

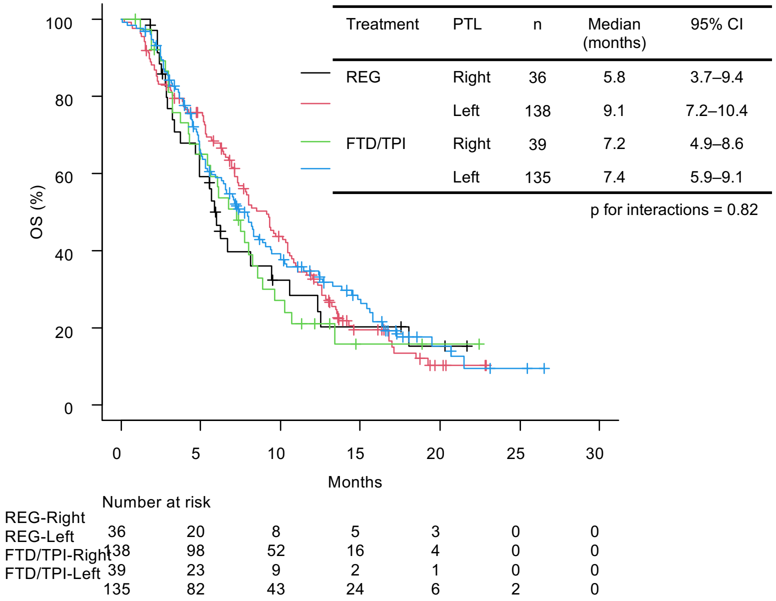

Supplemental Figure 3B

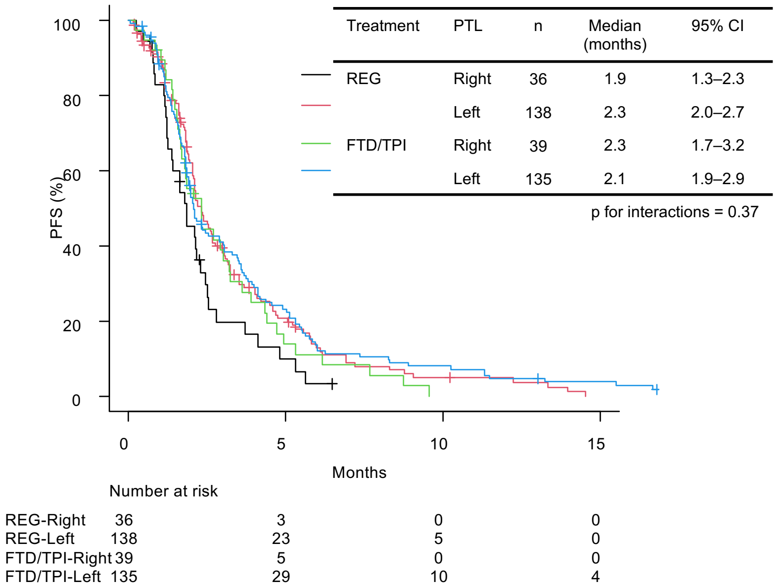

Supplemental Figure 3. (A) Kaplan-Meier curves of overall survival (OS) stratified by treatment group and primary tumor location (PTL) in the propensity score matching cohort. (B) Kaplan-Meier curves of progression-free survival (PFS) stratified by treatment group and PTL in the propensity score matching cohort. REG, regorafenib; FTD/TPI, trifluridine/tipiracil
