# Supplementary material for: Clinical Impact of Primary Tumor Location in Metastatic Colorectal Cancer Patients Under Later-Line Regorafenib or Trifluridine/Tipiracil Treatment
Source: Front Oncol. 2021 Jun 15;11:688709. doi: 10.3389/fonc.2021.688709 (PMC8239287; doi:10.3389/fonc.2021.688709)
Supplement: Supplementary file 2 [file Table_1.docx]

Supplemental Table 1. Subgroup analysis of disease control rate according to treatment group in each primary tumor location

|  | **Right** | | | **Left** | | |
| --- | --- | --- | --- | --- | --- | --- |
|  | **REG** | **FTD/TPI** | ***p* value** | **REG** | **FTD/TPI** | ***p* value** |
| **DCR** | 14 (25.5%) | 16 (28.1%) | 0.83 | 54 (34.4%) | 75 (30.0%) | 0.38 |

Abbreviations: DCR, disease control rate, REG, regorafenib; FTD/TPI, trifluridine/tipiracil

Supplemental Table 2. Univariate and multivariate analyses of overall survival (OS) in the propensity score-matched cohort

|  |  | **Univariate** |  | **Multivariate** |  |
| --- | --- | --- | --- | --- | --- |
| **Variable** | **Category** | **HR (95% CI)** | ***p* value** | **HR (95% CI)** | **p value** |
| **PTL** | Left *vs.* Right | 0.84 (0.63–1.14) | 0.27 | 0.97 (0.72–1.33) | 0.87 |
| **Treatment group** | FTD/TPI *vs.* REG | 1.00 (0.79–1.28) | 0.98 |  |  |
| **Age** | ≥ 65 *vs.* < 65 | 1.30 (1.02–1.66) | 0.037 | 1.44 (1.12–1.86) | 0.0047 |
| **Sex** | Female *vs.* Male | 0.96 (0.75–1.23) | 0.74 |  |  |
| **BMI** | ≥ 18.5 *vs.* 18.5 | 0.87 (0.62–1.23) | 0.44 |  |  |
| **ECOG PS** | PS2 v*s.* PS1 or 2 | 1.70 (0.97–2.98) | 0.063 | 1.79 (1.01–3.18) | 0.047 |
| **Surgery on primary resection** | Yes *vs.* No | 0.63 (0.48–0.85) | 0.0019 | 0.72 (0.54–0.97) | 0.031 |
| **Histology** | Others *vs.* well/mod | 0.88 (0.54–1.44) | 0.61 |  |  |
| **RAS status** | Mutant *vs.* Wild | 1.07 (0.85–1.33) | 0.58 |  |  |
| **Liver metastasis** | Yes *vs.* No | 1.52 (1.18–1.96) | 0.0013 | 1.43 (1.08–1.89) | 0.012 |
| **Lymph node metastasis** | Yes *vs.* No | 1.42 (1.11–1.82) | 0.0056 | 1.37 (0.99–1.88) | 0.057 |
| **–** | Yes *vs.* No | 0.83 (0.64–1.07) | 0.16 |  |  |
| **Peritoneal metastasis** | Yes *vs.* No | 1.76 (1.31–2.37) | < 0.001 | 1.78 (1.28–2.48) | < 0.001 |
| **Number of metastatic organ site(s)** | ≥ 3 *vs.* < 3 | 1.57 (1.21–2.04) | < 0.001 | 1.07 (0.74–1.55) | 0.71 |
| **Duration from initiation of 1st line chemotherapy** | ≥ 18 months *vs.* < 18 months | 0.60 (0.46–0.79) | < 0.001 | 0.61 (0.46–0.81) | < 0.001 |
| **Prior regimens** | ≥ 3 *vs.* < 3 | 0.92 (0.72–1.17) | 0.49 |  |  |

^＊^*P* values were calculated using the Cox proportional-hazards model.

Abbreviations: BMI, body mass index; ECOG PS, Eastern Cooperative Oncology Group performance status; IQR, interquartile range; RAS, rat sarcoma; REG, regorafenib; FTD/TPI, trifluridine/tipiracil.
